# The Association of Patent Ductus Arteriosus with Inflammation: A Narrative Review of the Role of Inflammatory Biomarkers and Treatment Strategy in Premature Infants

**DOI:** 10.3390/ijms232213877

**Published:** 2022-11-10

**Authors:** Yu-Jen Wei, Rosie Hsu, Yung-Chieh Lin, Tak-Wah Wong, Chung-Dann Kan, Jieh-Neng Wang

**Affiliations:** 1 Department of Pediatrics, National Cheng Kung University Hospital, College of Medicine, National Cheng-Kung University, Tainan 701401, Taiwan; 2 Institute of Clinical Medicine, College of Medicine, National Cheng-Kung University, Tainan 70403, Taiwan; 3 Department of Pediatrics, National Taiwan University Hospital, Taipei 100226, Taiwan; 4 Department of Dermatology, National Cheng Kung University Hospital, College of Medicine, National Cheng Kung University, Tainan 701401, Taiwan; 5 Department of Biochemistry and Molecular Biology, College of Medicine, National Cheng Kung University, Tainan 701401, Taiwan; 6 Center of Applied Nanomedicine, National Cheng Kung University, Tainan 701401, Taiwan; 7 Department of Surgery, Institute of Cardiovascular Research Center, National Cheng Kung University Hospital, College of Medicine, National Cheng Kung University, Tainan 701401, Taiwan

**Keywords:** ductus arteriosus, intrauterine inflammation, preterm infants, chorioamnionitis

## Abstract

Patent ductus arteriosus (PDA) is a common cardiovascular complication that complicates clinical care in the intensive care of premature infants. Prenatal and postnatal infections and the inflammation process can contribute to PDA, and intrauterine inflammation is a known risk factor of PDA. A variety of inflammatory biomarkers have been reported to be associated with PDA. Chorioamnionitis induces the fetal inflammatory process via several cytokines that have been reported to be associated with the presence of PDA and may have a role in the vascular remodeling process or vessel dilation of the ductus. On the other hand, anti-inflammatory agents, such as antenatal steroids, decrease PDA incidence and severity in patients born to those with chorioamnionitis. Proinflammatory cytokines, which are expressed more significantly in preterm neonates and chorioamnionitis, are associated with the presence of PDA. In this review, we focus on the pathogenesis of PDA in preterm infants and the role of biomarkers associated with the perinatal inflammatory process.

## 1. Introduction

A ductus arteriosus is a necessary cardiac structure in the fetal period, guiding fetal pulmonary blood flow, oxygenated by the placenta, to the aorta [1]. Closure of the ductus arteriosus is achieved by the contraction of the muscular media, which is the middle layer of the ductus composed of circumferential smooth muscle fibers [2,3]. The contraction of the media is triggered by high arterial oxygen content, a decline in pulmonary vascular resistance, and prostaglandin E2 in the circulation soon after birth [4,5]. In normal full-term neonates, functional closure of the ductus arteriosus occurs soon after birth; in contrast, delayed closure may occur in premature babies—the closure rate is inversely related to gestational age [6,7] because the muscular media of the ductus is inadequately developed and poorly responsive to high oxygen stimulation [4,8]. Persistent PDA in term babies is due to a deficiency of the muscular media in the wall of the ductus. Thus, PDA rarely closes spontaneously or even following pharmacological intervention in term babies due to an uncorrected anatomical defect [9]. Therefore, the principles of the management of PDA in term babies are different from premature infants. PDA has been reported to be associated with various prematurity-related complications [10,11,12,13,14,15,16,17,18,19,20,21]. Therefore, understanding different mechanisms that are involved in maintaining ductal tone is essential to comprehending why preterm infants have a high incidence of ductal patency, why some of them do not respond to treatment, and where to direct novel therapeutic approaches.

Maternal infections play a significant role in spontaneous preterm labor and birth as well as in related neonatal complications [22]. Previous reports demonstrated that infection episodes in the postnatal period are related to late ductal reopening and failed PDA closure [23], while a recent study only demonstrated a relatively higher risk without statistical significance [24]. Preterm infants may undergo a sustained inflammation process, a result of exposure to either prenatal or postnatal factors, and this may contribute to prematurity-related complications [25]. Studies also revealed the preterm delivery rate was increased during the COVID-19 pandemic, although the results are heterogeneous [26,27,28,29,30,31,32,33]. The rate of neonatal-related morbidities was also increased during the COVID-19 pandemic [29]. Therefore, managing prematurity-related complications is a crucial issue in this period.

The link between preterm labor, intrauterine inflammation, and PDA has been extensively studied in recent years, and seems to be generally agreed upon. However, studies have mainly focused on the clinical association. To date, there are limited systemic reviews investigating the immunologic and molecular mechanisms of this issue. The main goal of this review is to gain a better understanding of the role of intrauterine inflammatory biomarkers in PDA in premature infants.

## 2. Methods

This review was conducted to identify current evidence of the association between intrauterine inflammation and PDA in preterm neonates. The primary outcome is the failure of PDA closure, either failure to close spontaneously or medically. The secondary outcome is death before 36 weeks postmenstrual age, bronchopulmonary dysplasia (BPD), or organ damage.

The inclusion criteria included case–control studies or cohort studies related to preterm infants, PDA, and chorioamnionitis. Exclusion criteria included studies related to term infants (defined as gestational age > 37 weeks) or studies without a control group.

**Figure 1:**
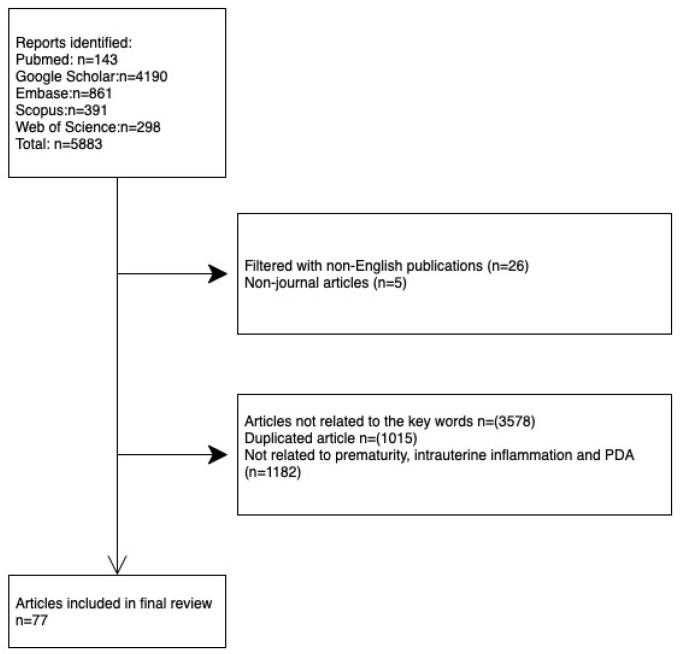
A PRISMA flow diagram of searching strategy of relevant articles.

A literature search in PubMed, Google Scholar, Embase, Scopus, and Web of Science was conducted using Boolean logic with the key words “chorioamnionitis” OR intrauterine infection” OR “intrauterine inflammation” OR “placental inflammation” AND “ductus arteriosus” OR “PDA” OR “patent ductus arteriosus”. Studies published between 1st January 1980 and 30th September 2021 were included. The included reports were filtered for duplicates and non-English publications. The resulting database was further filtered by the following exclusion criteria: (a) reports assessing term infants; (b) reports that did not focus on perinatal inflammation and PDA; (c) reports studying congenital anomalies, including congenital heart disease, chromosomal anomaly, genetic disorders, or metabolic disorder; and (d) reports only published in conference presentations. A PRISMA flow diagram is shown in Figure 1.

## 3. Results

A total of 5883 articles from the above databases were identified. Of them, 31 were excluded due to them being non-English journal articles. We then reviewed these articles and excluded the duplicates. A total of 4760 articles that were not related to the key words we searched for or not related to prematurity, ductus arteriosus, or intrauterine inflammation were also excluded. Finally, 77 articles were included for our review of prematurity and intrauterine inflammation-associated PDA.

### 3.1. The Role of Perinatal Infection and Inflammation in PDA

#### 3.1.1. Chorioamnionitis

Chorioamnionitis is defined clinically and histologically. Clinical chorioamnionitis may be defined by the Gibbs criteria [34], on the basis of maternal fever and two or more of the following additional criteria: maternal tachycardia, fetal tachycardia, uterine tenderness, foul odor of the amniotic fluid, and maternal leukocytosis. In 2016, the *Eunice Kennedy Shriver* National Institute of Child Health and Human Development (NICHD) provided updated evidence-based guidelines for the diagnosis of chorioamnionitis. The panel proposed replacing the term “chorioamnionitis” with “intrauterine inflammation or infection or both”, which should fit the following criteria: maternal fever (≥39.0 °C or repeatedly ≥38.0 °C with interval of 30 min) with one or more of the following: (1) fetal tachycardia (>160 bpm for 10 min or longer); (2) maternal white blood cells count >15,000 in absence of corticosteroids; (3) purulent fluid from the cervical os (cloudy or yellowish thick discharge confirmed visually on speculum exam); and (4) biochemical or microbiological amniotic fluid results consistent with microbial invasion of the amniotic cavity [35]. Histological chorioamnionitis may be defined by the infiltration of polymorphonuclear leukocytes in the placental membranes [36], or by the Salafia criteria [37] and the Blanc criteria [38]. Clinical and histological chorioamnionitis are not mutually inclusive, and, most often, clinical chorioamnionitis is not proven by histological findings due to the lack of amniotic fluid or placenta obtained for analysis.

A variety of pathogens may be associated with chorioamnionitis. However, the reported pathogen varies and may be different according to the definition of chorioamnionitis. Despite heterogenous reports of the incidences of isolated pathogens, the most commonly reported pathogen associated with chorioamnionitis and an adverse neonatal outcome was *Ureaplasma urealyticum* [39]. Other possible pathogens related to maternal infection include *E. coli*, Gram-positive streptococcus (GBS), and *Mycoplasma hominis* (Table 1) [34,40].

Histological chorioamnionitis has been associated with an increased risk of PDA and the failure of medical closure of PDA [41,42,43]. Combined clinical and histological chorioamnionitis also showed an increased risk of PDA (OR 1.75; 95% CI: 1.07–2.86); however, clinical chorioamnionitis without histological evidence was not found to be associated with increased risk (OR 1.28; 95% CI: 1.00–1.64) [42]. The incidence of PDA requiring surgical ligation did not differ between the chorioamnionitis group and the non-chorioamnionitis group in Park et al. in their recent meta-analysis [42].

Chorioamnionitis may induce the production of nitric oxide synthetase (NOS) and COX-2, resulting in the production of vasodilatory prostaglandins. Prostaglandins produced in the amniotic sac are normally inactivated by prostaglandin dehydrogenase released by the chorionic tissue. Infection of the chorion inhibits the activity of prostaglandin dehydrogenase [44]. However, COX-2 was not expressed in most of the cases unresponsive to indomethacin. The attenuation of COX-2 expression may be related to gestational ages. On the other hand, an increase in COX-1 expression in the umbilical artery in infants with intrauterine infection compared to those without intrauterine infection was demonstrated by Kim et al. [41]. The production of NOS and COX-1 may impede the indomethacin-induced ductal constriction in the postnatal period [42].

#### 3.1.2. Inflammation and PDA

Though there are a multitude of studies supporting the significant association between chorioamnionitis and PDA, the meta-analysis of Behbodi et al. [45] found a significant negative association after adjustment for confounding factors between gestational age and body weight. This was contradictory to the results from Park et al [42]. Behbodi et al. theorized that chorioamnionitis may play a protective role in PDA by facilitating lung maturation and decreasing the use of mechanical ventilation and surfactants [45]. Similar to the prior debate regarding the association between chorioamnionitis and chronic lung disease [46], the different baseline characteristics of the chorioamnionitis-exposed group and control group should be taken into account during the analysis of chorioamnionitis and PDA. Differences in gestational age and body weight between the chorioamnionitis-exposed group and the control group significantly correlated with the effect size of the association between PDA and chorioamnionitis. Notably, the infants exposed to chorioamnionitis were of significantly lower gestational age and body weight [46]. In fact, previous studies provided evidence that chorioamnionitis and PDA are inversely related to gestational age [47,48,49,50,51,52]. Current research primarily focuses on the association between PDA and inflammation in preterm infants. This issue in term infants is poorly discussed. Otsubo et al. presented the association of inflammatory biomarkers with the gestational age, and the incidence of PDA. There were no infants older than GA 32 weeks diagnosed with PDA [53]. Park et al. reported an increased risk of PDA in infants born with chorioamnionitis. Although this meta-analysis consisted of studies including populations mainly born with GA less than 34 weeks, some studies including near-term populations were also included. In this review, the gestational age was not shown to be a covariate for the incidence of PDA [42].

Pietrasanta et al. echoed the concern about the confounding factor of gestational age [54]. However, Pietrasanta et al. proposed that instead of looking at gestational age as a confounding factor or an independent variable, it should be considered as an “intermediate” variable. Investigation of the independent effect of intrauterine inflammation on neonates should be performed with a direct comparison between preterm neonates with intrauterine inflammation and preterm neonates with other underlying circumstances [54]. This proposal is based on the fact that gestational age is the consequence of any underlying cause of preterm birth, including intrauterine inflammation. In the prospective cohort study, the authors investigated the role of gestational age in the effect of intrauterine inflammation on various outcomes of preterm neonates, independently of other covariates (maternal age, ethnicity, antenatal steroid, sex, birth body weight, and induced delivery in the absence of preterm labor). To do so, two multivariate analyses were performed, one with the mentioned covariates but without gestational age and one with gestational age. Prior to the inclusion of gestational age, histological chorioamnionitis was only significantly associated with the increased risk of retinopathy of prematurity (ROP), and histological chorioamnionitis with funisitis was associated with an increased odds ratio of respiratory distress syndrome (RDS), BPD, early-onset sepsis (EOS), ROP, intraventricular hemorrhage (IVH), and PDA. After the inclusion of gestational age, histological chorioamnionitis was also found to be a protective factor against mechanical ventilation and the need for exogenous surfactants, and histological chorioamnionitis with funisitis was no longer associated with RDS, BPD, ROP, IVH, and PDA [54].

In the last 5 years, studies’ results have still differed regarding the association between chorioamnionitis and PDA. Two single-center studies demonstrated a lack of association between clinical chorioamnionitis and PDA [55,56], compatible with the findings of Park et al [42]. However, in a recent national U.S. cohort study, clinical chorioamnionitis was associated with increased odds of PDA after adjustment for gestational age [57]. The discrepancy between study results may be due to the wide variety of diagnoses and treatments of clinical chorioamnionitis in clinical practice [58] and the discordance between the histopathology and clinical diagnosis of chorioamnionitis [59]. A prospective single-center study was conducted in Turkey and found no differences in hemodynamically significant PDA, BPD, necrotizing enterocolitis (NEC), and mortality among the placentas categorized as normal placenta, or with vasculopathy and chorioamnionitis by histopathology [60]. Notably, hemodynamically significant PDA was specifically required in the particular study in Turkey, a requirement absent in the recent meta-analyses [42,45]. On the other hand, a population-based cohort study in Korea found a protective role of histological chorioamnionitis in the development of symptomatic PDA in infants of 26–29 gestational weeks, an effect not found in infants of 22–25 gestational weeks [61]. The cohort study supported the results of Behbodi et al [45]. Therefore, whether chorioamnionitis is an independent risk factor of PDA is yet to be determined and may require large, prospective studies with the relationship of chorioamnionitis and PDA as the main research question as well as carefully selected target populations for comparison.

### 3.2. Antenatal Steroid Usage and Its Impact on PDA Closure

Antenatal steroid usage is a common medical practice in maternal women expecting a preterm birth as steroids accelerates the maturation of the fetuses’ lungs and reduces the risks of RDS. In theory, corticosteroids could activate and/or worsen infection due to their immunosuppressive effect. Owing to the immunosuppressive effect of corticosteroids, guidelines for antenatal steroids list chorioamnionitis as a contraindication [62,63]. Conversely, studies found an association of antenatal steroid usage with the decreased risk of PDA, particularly among patients with histological chorioamnionitis and less severe chorioamnionitis [42,52,61,64,65,66]. Antenatal steroids may reduce the sensitivity of the ductus arteriosus to prostaglandin and oxygen and affect the synthesis of prostaglandin [67,68,69]. Antenatal steroids may also inhibit the induction of iNOS and COX-2 in the presence of inflammation, resulting in ductal constriction [70].

Nonetheless, recent systematic reviews and meta-analyses did not confirm a statistically significant relationship between antenatal steroid usage and PDA [45,69]. Notably, a wide variance in steroid choice and duration of therapy was seen in the studies included in the two systematic reviews and meta-analyses [45,69], suggesting a need for randomized control studies or prospective studies with a strict protocol. Furthermore, among infants of <26 gestational weeks, antenatal steroids could only cause the constriction of the ductus arteriosus when prostaglandin production had been eliminated, which suggests that antenatal steroids have different roles at different gestational ages [71].

The effect of postnatal corticosteroids on the decrease in the incidence of PDA is also of interest, and has been preliminarily supported by the two Cochrane meta-analyses on postnatal corticosteroids for the prevention of chronic lung disease [72,73]. However, gastrointestinal bleeding, intestinal perforation, hyperglycemia, and hypertension were important adverse events that increased with the use of postnatal corticosteroids [63,64]. More studies should be commenced to investigate the relationship between postnatal corticosteroid use and PDA.

### 3.3. Potential Biomarkers Involved in PDA Pathogenesis

The fetal inflammatory response induced by chorioamnionitis has also been extensively studied. Fetal inflammatory response syndrome (FIRS) is defined as the elevation of interleukin-6 (IL-6) levels, >11 pg/mL, in the umbilical cord [39]. In FIRS, the fetal immune system is activated, and this has been associated with a higher risk of neonatal morbidity and mortality, including IVH, BPD, periventricular leukomalacia, and cerebral palsy [39,40,74,75,76,77,78]. Similarly, high levels of inflammatory markers in the umbilical cord, including IL-6, -8, -10, -12, growth/differentiation factor 15 (GDF-15), monocyte chemoattractant protein-1 (MCP-1/CCL2), and macrophage inflammatory protein-1α (MIP-1α/CCL3), were associated with the development and persistence of PDA [53,79]. IL-6, -8, and -12 are proinflammatory markers that have been associated with pulmonary morbidity and vascular remodeling [74,79]. IL-6 is increased during the active phase of delivery, significantly elevated in preterm birth compared to term delivery, and increased in neonates exposed to histologic chorioamnionitis [80,81]. Increased levels of IL-6 stimulate the primary decidual and amnion cells and induce a concentration-related increase in prostaglandin [82]. MCP-1/CCL2 is a key chemokine that facilitates the migration and infiltration of monocytes and macrophages [83], while MIP-1α/CCL3 is secreted by macrophages and recruits other macrophages, lymphocytes, and eosinophils [84]. The potential biomarkers that may be involved in the pathogenesis or risk of PDA are summarized in Table 2. In normal physiology, the increased recruitment of monocytes and macrophages should increase levels of platelet-derived growth factors essential for the migration and proliferation during the remodeling process. However, the association of increased chemotaxis chemokines with PDA is an indication of a more complex interaction during the closure of DA that has yet to be elucidated. Nonetheless, intrauterine inflammation may not always induce a fetal inflammatory response. If a fetal inflammatory response is induced, funisitis and chorionic vasculitis may be seen in the placenta [85]. Behbodi et al. showed that the presence of funisitis combined with chorioamnionitis did not significantly change the odds of PDA compared to chorioamnionitis without the presence of funisitis, arguing against the etiopathogenic role of the fetal inflammatory response for PDA [45].

A recent review emphasized the theory of unbalanced inflammatory regulation mechanisms in preterm infants. These premature infants are exposed to either environmental or endogenous risks of inflammation and are more likely to undergo an unresolved inflammatory process, resulting in sustained inflammation [25]. Maternal infection is associated with increased proinflammatory cytokine production, including TNF-α, IL-1β, and IL-6. Prostaglandin E increases the expression of IL-15 mRNA in cultured ductal smooth muscle and attenuates platelet-derived growth factor. IL-15 inhibits the physiologic vascular remodeling process and contributes to the pathogenesis of the persistent ductus in animal models [86,87]. IL-17 increases platelet adhesion and may contribute to ductus obliteration in the process of ductus closure. However, IL-17 also stimulates prostaglandin production. IL-17 may play different roles in vascular remodeling or dilation depending on its concentration [88,89].

### 3.4. Pharmacological Treatment in PDA Closure

In recent years, medical treatment for PDA has been reserved for hemodynamically significant PDA (hsPDA). Indomethacin and ibuprofen are common pharmaceutical choices in the treatment of hsPDA, while acetaminophen is a popular alternative choice of drug. Although chorioamnionitis may be a risk factor for PDA, current evidence does not suggest an alternative treatment strategy of PDA treatment in infants born with maternal chorioamnionitis. In addition, the proportion of infants needing secondary surgical ligation of PDA was not increased [61]. The prostaglandin-H2 synthetase (PGHS) enzyme system has two active sites, COX and peroxidase (POX) [90]. Both indomethacin and ibuprofen are non-steroidal anti-inflammatory drugs (NSAID) that induce the closure of PDA by blocking the production of prostaglandin via inhibiting COX [90,91,92]. Acetaminophen exerts its effect on PDA through the direct inhibition of prostaglandin synthetase via the POX region and by decreasing the synthesis of prostaglandins [90].

#### 3.4.1. Indomethacin

Indomethacin was the first drug used for PDA, with a closure rate of around 70~85% [90,93,94]. Since the first use of indomethacin prophylactically, the incidence of severe IVH, hsPDA, and severe pulmonary hemorrhage, and the necessity of surgical ligation of PDA, have reduced [91,93,95,96], but without a significant effect on mortality or long-term neurodevelopmental outcomes [96]. However, the drug has been associated with side effects such as renal impairment, with presentations of acute or chronic renal failure, oliguria, proteinuria [97], NEC, intestinal perforation, and platelet dysfunction [93,98,99]. This detrimental effect on renal function is due to the inhibition of prostaglandin synthesis, causing vasoconstriction and a decrease in renal blood flow and glomerular filtrates [100]. The renal impairment is typically transient but may be complicated by oliguria, fluid overload, and electrolyte abnormalities [100].

#### 3.4.2. Ibuprofen

Ibuprofen was introduced into the clinical practice of treating hsPDA in light of the side effects seen with indomethacin. The mechanism, efficacy, duration, and timing of both ibuprofen and indomethacin are similar, but ibuprofen is superior in its reduced impact on renal function and NEC [91,93,100,101]. Currently, ibuprofen is the first choice of drug for hsPDA treatment. In fact, Mitra et al. found that a high dose of oral ibuprofen (15 to 20 mg/kg followed by 7.5 to 10 mg/kg every 12–24 h for a total of three doses) was associated with the best odds of hsPDA closure [102]. This finding was comparable to a Cochrane review [101] in which intravenous ibuprofen was significantly less efficacious than oral ibuprofen in the closure of PDA. The slower absorption rate and longer half-life of oral ibuprofen may prolong its time in contact with PDA, leading to a higher efficacy of oral ibuprofen compared to intravenous ibuprofen [103]. Pacifici et al. also recognized the contribution of CYP2C9 and CYP2C8, subfamilies of the cytochrome P450 complex, to the metabolism of ibuprofen [103]. The activity of CYP2C9 is low at birth, surges during the first week of life, and reaches about one-third of the adult value at the end of the first month [104], explaining the significant increase in ibuprofen clearance and failed PDA closure with increasing postnatal age [92].

#### 3.4.3. Acetaminophen

Recently, acetaminophen has attracted increased attention in the treatment of PDA and is widely used in cases of contraindicated ibuprofen or indomethacin and/or the failure of medical closure [93]. Meta-analysis showed that oral paracetamol better promoted primary PDA closure than a placebo [105]. Terrin et al. revealed the similar efficacy rate and safety profile of paracetamol and ibuprofen [106]. Similar to ibuprofen and indomethacin, greater efficacy of paracetamol is observed when treatment is started in the first week of life due to the physiological decrease in circulating levels of prostaglandins as postnatal age increases [106]. An oral dose of paracetamol has been shown to be more efficacious than the intravenous route, perhaps due to the same reason as the efficacy of ibuprofen [103,106]. A recent systematic review also indicated no significant difference between paracetamol and ibuprofen and between paracetamol and indomethacin in PDA closure [105]. The mean number of hours until PDA closure and the proportion of GI bleeding and hyperbilirubinemia were significantly reduced with paracetamol compared with ibuprofen [105]. The adverse effects of acetaminophen include hepatotoxicity presenting with a transient increase in liver enzymes [90,107]. Hepatotoxicity following exposure to acetaminophen may be due to the dosage range used for PDA closure, which is twice as high as the authorized recommended dosage for term neonates [94].

#### 3.4.4. Resistance to Pharmacological Treatment

Genetic variance may play a role in resistance to the current PDA pharmacological treatment strategy. Cytochrome P450 enzymes, particularly CYP2C9 and CYP2C8, may contribute to the clearance of indomethacin and ibuprofen. Carriers of variant alleles of these enzymes exhibit lower clearance and may exhibit higher physiological concentrations after drug administration, potentially contributing to a greater clinical effect. Research has shown that genes involving prostaglandin and NO action or synthesis are related to non-responsiveness to pharmacological treatment, such as *SLCO2A1*, *PTGS2*, and *NOS3* [108].

#### 3.4.5. Drugs in the Future

Pharmacological treatment for PDA closure is not without its side effects, even with acetaminophen as an alternative. A promising possibility of the application of nanomedicine on PDA closure is on the horizon. The concept of nanomedicine has been applied clinically in the fields of oncology and infectious diseases for more than two decades [109,110]. The first FDA-approved nano-drug Doxil^®^, approved in 1995, was a liposomal doxorubicin targeting tumor cells [109]. Nanocarriers may act as transport vehicles that allow local targeted drug delivery, such as statins, to the site of action [111]. Since then, new prospects for nanomedicine application in the maternal–fetal and cardiology fields have been investigated. The use of oxytocin receptor-targeted liposomes, nanoparticles built from phospholipid bilayers, and those loaded with nifedipine, salbutamol, or rolipram successfully abolished human myometrial contractions in vitro [112]. The immunoliposomes conjugate to the oxytocin receptor antibodies that target the oxytocin receptor on the pregnant uterus. The oxytocin receptor-targeted liposomes loaded with indomethacin were also effective in reducing preterm birth in mice [112]. Previous studies reported applications of ανβ3-targeting, fumagillin-carrying nanoparticles in rat models with a resulting reduction in microvessels in the aorta and reduction in neovascular signals when combined with oral atorvastatin [113,114]. Another study used biodegradable polymeric nanoparticles to envelope sirolimus that were endocytosed, and inhibited the viability and proliferation of the human coronary smooth muscle cells and endothelial cells in vitro under a short initial burst release followed by a slow continuous release period [115]. Oduk et al. experimented with VEGF-containing nanoparticles that prolonged the exposure to a low dose of VEGF, increasing the angiogenic and therapeutic potency of VEGF for the treatment of myocardial infarction [116]. With the success of the in vitro studies, we can extrapolate that nano-encapsulated drugs such as indomethacin may hopefully be able to target the COX2 and POX active sites, while VEGF-containing nanoparticles may lead to the cascade of remodeling process necessary for anatomical PDA closure.

The variants in the gene associated with a risk of PDA could be a potential target for a novel therapeutic approach, such as CYP8A1 and CYP1B1. PTGER4 is a predominant prostaglandin receptor in the DA and may be another potential target protein [117]. Phosphodiesterase inhibitor may cause ductus dilation, and PDE1B, PDE3B, and PDE5A activators may induce ductus closure [108].

## 4. Conclusions

Intrauterine infection may result in the inflammatory process in fetuses and infants. This inflammatory response may persist in premature infants due to an unbalanced immune system and may result in sustained inflammation in these infants, causing prematurity-related morbidity. Animal models and clinical research showed that the expression of proinflammatory cytokines during this process contributes to these complications, and potentially contributes to persistent ductus patency. Further investigation on these inflammatory pathways is needed to clarify the detailed mechanism.

## Figures and Tables

**Table 1:** Maternal pathology, association of pathogens and neonatal outcome.

Reference	Maternal Pathology	Pathogens	Outcome
Gomez et al. [39]	Clinical or histological chorioamnionitis Microbial invasion of amniotic cavity	*U. urealyticum* (70%) *M. hominis* (24%) *Fusobacterium* (9%) *Strp. Viridans* (9%)	Elevation of IL-6 in fetus
Gibbs et al. [34]	Intraamniotic infection	*U. urealyticum* (47%), *M. hominis* (31%), *B. bivius* (29%), *G. vaginalis* (24%) GBS (15%), *E. coli* (8%)	Poor neonatal outcomes in intraamniotic fluid with *E. coli* or GBS
Chang et al. [40]	Histological chorioamnionitis	*U. urealyticum*	*U. urealyticum* was more frequent in infant with BPD

**Table 2:** Potential biomarkers involved in PDA pathogenesis.

Biomarker	Potential Pathological or Clinical Role That May Relate to Perinatal Inflammation and PDA	References
TNF-α	Mediators in the early inflammatory response	[25]
IL-1	Mediators in the early inflammatory response Risk of preterm birth	[25,84]
IL-6	Mediators in the early inflammatory response Risk of preterm birth Clinically related to persistent PDA	[25,39,74,79,84]
IL-8, IL-10, MIP-1α	Related to persistent PDA Clinical risk of preterm birth	[79,84]
IL-15	Attenuates smooth muscle cell proliferation Involved in atherogenesis	[86,87]
IL-17	Risk of preterm birth Involved in vascular remodeling and prostaglandin expression Increases platelet aggregation	[84,88]
GDF-15	Related to persistent PDA Associated with tissue hypoxia, inflammation, acute injury, and oxidative stress.	[79]
ΜCP-1	Clinically related to persistent PDA Regulates migration and infiltration of monocytes and macrophages Risk of preterm birth Related to thrombus formation	[79]
PGDH	Risk of preterm birth	[84]

## Data Availability

No new data were created or analyzed in this study. Data sharing is not applicable to this article.
